# Genome-wide insights on gastrointestinal nematode resistance in autochthonous Tunisian sheep

**DOI:** 10.1038/s41598-021-88501-3

**Published:** 2021-04-29

**Authors:** A. M. Ahbara, M. Rouatbi, M. Gharbi, M. Rekik, A. Haile, B. Rischkowsky, J. M. Mwacharo

**Affiliations:** 1 Small Ruminant Genomics, International Centre for Agricultural Research in the Dry Areas (ICARDA), Addis Ababa, Ethiopia; 2 Department of Zoology, Faculty of Sciences, Misurata University, Misurata, Libya; 3 Laboratory of Infectious Animal Diseases, Zoonosis and Sanitary Regulation, University of Manouba, Manouba, Tunisia; 4 Institution of Agricultural Research and Higher Education, National School of Veterinary Medicine of Sidi Thabet, Sidi Thabet, Tunisia; 5 Animal and Veterinary Sciences, Scotland’s Rural College (SRUC) and Centre for Tropical Livestock Genetics and Health (CTLGH), The Roslin Institute, Midlothian, UK

**Keywords:** Evolutionary genetics, Molecular evolution, Population genetics, Evolution, Genetics, Agricultural genetics, Animal breeding, Genomics, Population genetics

## Abstract

Gastrointestinal nematode (GIN) infections have negative impacts on animal health, welfare and production. Information from molecular studies can highlight the underlying genetic mechanisms that enhance host resistance to GIN. However, such information often lacks for traditionally managed indigenous livestock. Here, we analysed 600 K single nucleotide polymorphism genotypes of GIN infected and non-infected traditionally managed autochthonous Tunisian sheep grazing communal natural pastures. Population structure analysis did not find genetic differentiation that is consistent with infection status. However, by contrasting the infected versus non-infected cohorts using ROH, LR-GWAS, F_ST_ and XP-EHH, we identified 35 candidate regions that overlapped between at least two methods. Nineteen regions harboured QTLs for parasite resistance, immune capacity and disease susceptibility and, ten regions harboured QTLs for production (growth) and meat and carcass (fatness and anatomy) traits. The analysis also revealed candidate regions spanning genes enhancing innate immune defence (SLC22A4, SLC22A5*,* IL-4, IL-13), intestinal wound healing/repair (IL-4, VIL1, CXCR1, CXCR2) and GIN expulsion (IL-4, IL-13). Our results suggest that traditionally managed indigenous sheep have evolved multiple strategies that evoke and enhance GIN resistance and developmental stability. They confirm the importance of obtaining information from indigenous sheep to investigate genomic regions of functional significance in understanding the architecture of GIN resistance.

## Introduction

Small ruminants (sheep and goats) make immense socio-economic and cultural contributions across the globe. However, gastrointestinal nematode (GIN) infections pose the main constraint to grazing small ruminants^1, 2^. The control and treatment of GIN infections is estimated to cost tens of billions of dollars worldwide^3^ and has traditionally relied on chemotherapeutics but, their extended use has resulted in economic losses and raised concerns of environmental health, food safety, and the development of resistance in parasites for all the major groups of anthelminthic drugs^1, 4, 5^. The evolution of anthelmintic resistance and changes in climate, land-use and farming practices are likely to alter the geographic distribution and infection patterns of parasites and their impacts, calling for the development of sustainable control strategies^2, 6^.

Evidence for host genetic variability for GIN resistance has been observed in ruminant livestock suggesting selective breeding is a feasible control option^7^. Indicator traits, such as faecal egg count (FEC), antibody assays, packed cell volume (PCV) and FAMACHA scores have been used to identify resistant and resilient animals^8, 9^. However, the mechanisms underlying genetic differences in resistance to GIN infections remain poorly understood, with resistance being a physiologically complex trait that develops over time, and indicator traits measured at specific time points may fail to represent all the pathways involved. Most often, quantifying resistance has been through artificial challenge using variable doses of larvae with which the rate, time and specificity of infection are controlled. However, traditional communal grazing management practised in smallholder mixed farming, nomadic and pastoral systems predominate in the (sub)tropics. In these systems, multiple nematode species account for natural infections which occur gradually, and findings from single infection studies may not mirror the patterns of infection encountered in these traditional systems^10^. There are reports showing between- as well as within-breed variation in resistance to GIN in small ruminants^11–13^. These observations and the genetic fragmentation observed in most breeds, implies that information derived from one breed cannot be extrapolated to another but needs to be validated for individual breeds^5^.

Advances in genomic technologies offer the opportunity to investigate the nature of genetic variation underlying complex traits^14^. The investigation and discovery of putative candidate genes, genomic and regulatory variants underpinning GIN resistance may provide a better understanding of the underlying molecular mechanisms and accelerate genetic gains in breeding programs. Genome-wide scans using single nucleotide polymorphisms (SNPs) have identified candidate regions, genes and QTLs putatively associated with GIN resistance on almost all ovine chromosomes^11, 15–19^. Most of the regions lie within and/or in proximity to MHC II and the interferon family of genes including the multitude of genes activated following interferon exposure^20, 21^, that are components of the immune response. Other QTLs for GIN resistance implicate other mechanisms, including innate and acquired immune response, gastric mucosal protection and haemostasis pathways^18^. Here, we utilise genome-wide autosomal genotype data generated with the Illumina Ovine 600 K SNP BeadChip and bioinformatic analysis, to conduct genome-wide screens in the hope of identifying regions and loci, linked to individual animal variability to GIN resistance in traditionally managed autochthonous Tunisian sheep.

## Materials and methods

### Study cohorts, sample collection, DNA extraction and genotyping

This study required no ethical approvals as all the samples were procured from slaughterhouses and in the presence of a veterinarian. In total, 309 blood samples were collected from indigenous sheep brought for slaughter at eight commercial slaughterhouses (Tunis (Tunis abattoir), Ariana (Ariana abattoir), Bizerte (Bizerte, Mateur and Sajnène abattoirs), Béja (Béja abattoir) and Jendouba (Jendouba and Tabarka abattoirs)) in northern Tunisia where GIN infections present one of the main constraints to small ruminant production^22^. The blood samples were collected using EDTA vacutainers by puncture of the jugular vein. DNA was extracted from each blood sample using WIZARD Genomic DNA Purification Kit (Promega, Madison, WI, USA) and then stored at − 80 °C. Out of the 309 samples, 101 were selected and assigned to two extreme groups based on GIN infection rates i.e., Non-infected (n = 58) and Infected (n = 43), based on faecal egg counts (FEC). FEC were estimated from 5 grams of stool by concentration floatation followed by the McMaster technique and the eggs per gram calculated to estimate the degree of infection^23^. The FEC readings ranged from zero (0) to 3800 eggs per gram. All individuals reporting an FEC = 0 were classified as non-infected and those with FEC > 100 were classified as infected.

Information on the grazing pattern and history of anthelmintic use was obtained from animal owners before slaughter. From faecal examinations, eggs of gastro-intestinal helminths (including nematodes and cestodes), Eimeria spp., Trichuris spp. and Nematodirus spp., were identified in the study individuals^23^ suggesting infections from multiple GIN species. The DNA samples were genotyped with the Illumina 600 K SNP BeadChip (Illumina Inc., San Diego, CA, USA) at GeneSeek Neogen Genomics, Lincoln NE, USA. The BeadChip comprises 606,006 probes that target genome-wide SNPs, among which 577,401 are autosomal, 27,314 are on the X chromosome and 1291 are unassigned.

### Data quality control and screening

The 606,006 raw SNP genotypes were processed for quality control with PLINK1.9^24^. The following criteria was used: (1) one individual was randomly selected from one pair of highly related animals with an identity-by-state score (IBS) of greater than 0.99, (2) SNPs with minor allele frequencies (MAF) of no less than 0.01 were retained, (3) individuals and SNPs with call rates lower than 90% and 95%, respectively were discarded and (4) all unmapped SNPs and those on the sex chromosomes were excluded. This generated a dataset of 540,528 autosomal SNPs and 92 samples (Infected = 41; Non-infected = 51). This dataset was subjected to LD pruning using the parameters 50 5 0.5 representing window size, step size and *r*^2^ threshold, respectively resulting in 335,070 SNPs that were used for population structure and phylogenetic analysis.

### Estimation of genetic diversity

Expected (H_E_) and observed (H_O_) heterozygosity, effective population size (N_E_), and patterns of LD decay were investigated for the two cohorts (infected and non-infected). H_E_ and H_O_ were calculated using PLINK v1.9. Pair-wise LD was evaluated using the correlation coefficient (*r*^*2*^) between alleles at two separate SNP loci using PLINK v1.9 with default settings. Following Sved^25^, N_E_ over generation time was estimated with the equation N_Et_ = (1/4c) (1/*r*^*2*^ − 1), where N_Et_ is the effective population size t generations ago (t = 1/2c); *r*^2^ is the LD between pairwise SNPs; and c is the genetic distance in Morgan between pairs of SNPs.

For each cohort, two measures of inbreeding were calculated; (1) SNP based inbreeding coefficient (*F*) calculated with PLINK v1.9 and (2) runs of homozygosity (ROH) based inbreeding coefficient (*F*_ROH_). For the latter, ROH streatches were first computed using the “detectRUNS” package in R^26^. The *F*_ROH_, was computed as the ratio of the total length of ROH to the length of autosomes (2.45 Gb)^27^.

Three estimates of ROH were calculated taking into account three genomic distance categories, ROH < 5 Mb, 5 Mb < ROH < 10 Mb, and ROH > 10 Mb, indicative of ancestral (more than ten generations), middle (5–10 generations), and recent (within five generations) inbreeding, respectively^28^. For the calculation, ROHs were identified for each individual using PLINK v 1.9 with the following parameters: (1) the minimum number of SNPs in a sliding window was 50; (2) the minimum ROH length was set to 1 Mb to eliminate the impact of strong LD; (3) each ROH spanned a minimum of 80 consecutive SNPs; (4) one heterozygous and five missing calls per window were allowed to avoid false negatives that may arise due to occasional genotyping errors or missing genotypes; (5) the minimum SNP density was set at one SNP every 100 kb, and the maximum gap between consecutive SNPs was set to 1 Mb.

### Population structure analysis

The 335,070 SNPs were used to infer genetic structure and divergence using three methods. Principal component analysis (PCA) was performed using PLINK v1.9 and the first two PC’s were plotted to visualize individual relationships. The proportion of shared ancestry between individuals was inferred with the unsupervised mode of ADMIXTURE tool v1.30^29^. This mode does not assume any background information on the number and frequency of alleles in ancestral populations. The ADMIXTURE tool was run with values of *K* increasing gradually from 1 to 6, to derive cross-validation (CV) errors. The lowest value of the CV error indicates the most likely number of ancestral populations. Five runs were performed for each *K*. To test for consistency in the ADMIXTURE results, we repeated the ADMIXTURE runs using three datasets of 6,000 SNPs, each drawn at random without replacement from the 335,070 SNPs. The pairwise allele sharing distance (ASD)^30^ was computed using the program “*asd*”. A distance matrix of all pairwise ASD dissimilarities, calculated as *1-ASD*, was generated and subjected to hierarchical clustering with the Neighbour-Joining algorithm to yield an individual clustering dendrogram. ASD calculation does not require estimates of allele/genotype frequencies making it valid when sample sizes are small. It is also suitable for detecting outliers and is robust to high LD among SNPs.

### Detection of selection signatures and association analysis

To investigate the molecular genetic basis underlying natural variation in GIN infection, we investigated genome-wide distributions of ROH in each individual in the infected and non-infected cohorts using PLINK v1.9. For this analysis, we used the 540,528 SNPs that passed the quality thresholds. Using the “detectRUNS”, we counted the number of times a given SNP occurred in the identified ROH in each cohort and presented a Manhattan plot of all the tested SNPs against their autosomal positions. The most frequently observed SNPs in ROHs occurring at the top 25% of the empirical distribution were taken as the most significant loci underlying an ROH under selection. To identify regions of ROH that are likely associated with variations in GIN infection, we compared the ROH regions between the infected and non-infected cohorts and identified the ones that were specific to the non-infected cohort.

We performed the logistic regression (LR) GWAS, F_ST_ and XP-EHH analyses to investigate further the genome regions associated with variation in GIN infection. LR-GWAS was performed with PLINK v1.9 using the non-infected cohort as the test sample and the infected cohort as the control. To obtain the 99% confidence intervals for the estimated parameters, the “-ci 0.99” and “-covar” options were invoked and Fisher’s exact test was used to generate the p-values considering location, breed, sex, and age as covariates. The raw p-values were subjected to Bonferroni correction to control the likelihood of any false positive results among the markers identified to be under selection. The corrected p-values were standardized and the − Log 10 (p-value) of 4.25 (the top 0.001) was set as the cut-off threshold to identify candidate regions. The estimations were summarized in 200 Kb window sizes and the genes falling within the candidate regions were identified. The package ’qqman’ in R version 3.5.1 was used to generate the Manhattan plot.

The population differentiation statistic, *F*_*ST*_, was used to investigate regions of the genome that have diverged between the two cohorts. The unbiased pairwise *F*_ST_^31^ was computed using the HIERFSTAT Package of R^32^ using a window size of 200 Kb and a window-step size of 100 Kb. Windows with less than five SNPs were excluded from the analysis. The *F*_ST_ values were then standardized by Z transformation following Ahbara et al.^33^. Windows falling within the top 0.001% of the *F*_ST_ values in each chromosome were considered the putative candidates under divergent selection.

The cross-population extended haplotype homozygosity (XP-EHH)^34^ was used to compare expected haplotype homozygosity (EHH) and integrated haplotype score (iHS) between the two cohorts to detect selection and its direction. XP-EHH scans SNPs that are homozygous in one population but polymorphic in the other through pairwise comparison of EHH scores. Positive XP-EHH scores indicate selection in the test sample, while negative scores indicate selection in the control. The XP-EHH scores were estimated as:

$${\text{XP}}-{\text{EHH}}=ln\left({\frac{{I}_{A}}{{I}_{B}}}\right)$$

where *I*_*A*_ is the integrated EHH value of the test population and *I*_*B*_ is the integrated EHH value of the reference population. Haplotype phasing was inferred for each cohort simultaneously on all SNPs using BEAGLE v3.3.1^35^. The XP-EHH test was performed with the “rehh” package of R^36^ and the raw XP-EHH scores were standardized to a distribution with zero mean and unit variance. Selection candidates were considered as the regions contained in any of the 200 Kb windows with a significance threshold of p < 0.001; this equates to an XP-EHH value of 4 at the default settings of “rehh” estimation.

### Functional annotation of candidate regions

The candidate regions identified by ROH were analysed and the ones that were specific to the non-infected cohort identified. We also analysed the ROH regions of the non-infected cohort, LR-GWAS, F_ST_ and XP-EHH candidate regions and the ones that overlapped between at least two approaches were identified and merged using Bedtools v.2.28.0^37^. Genes that were spanned by the ROH (non-infected cohort) and overlapping candidate regions were retrieved using the Biomart/Ensembl (http://www.ensembl.org/biomart) tool based on the Ovine v3.1 reference genome assembly. The set of genes identified in the candidate regions were assessed for biological enrichment gene ontology (GO) and KEGG Pathway (www.kegg.jp/kegg/kegg1.html) terms compared to the full list of *Ovis aries* autosomal protein-coding genes with the functional annotation tool in DAVID v6.8^38^ using *O. aries* as the background species. We also mapped the ROH (non-infected) and overlapping candidate regions with those reported in the sheep quantitative trait loci (QTL) database Release 42 (QTLdb; https://www.animalgenome.org/cgi-bin/QTLdb/OA/summary) to identify overlapping QTLs, which may suggest associations with response to parasite infections. To provide further biological interpretation, gene functions were determined from the NCBI database (http://www.ncbi.nlm.nih.gov/gene/) and review of literature.

## Results

### Genetic diversity estimates

**Figure 1:**
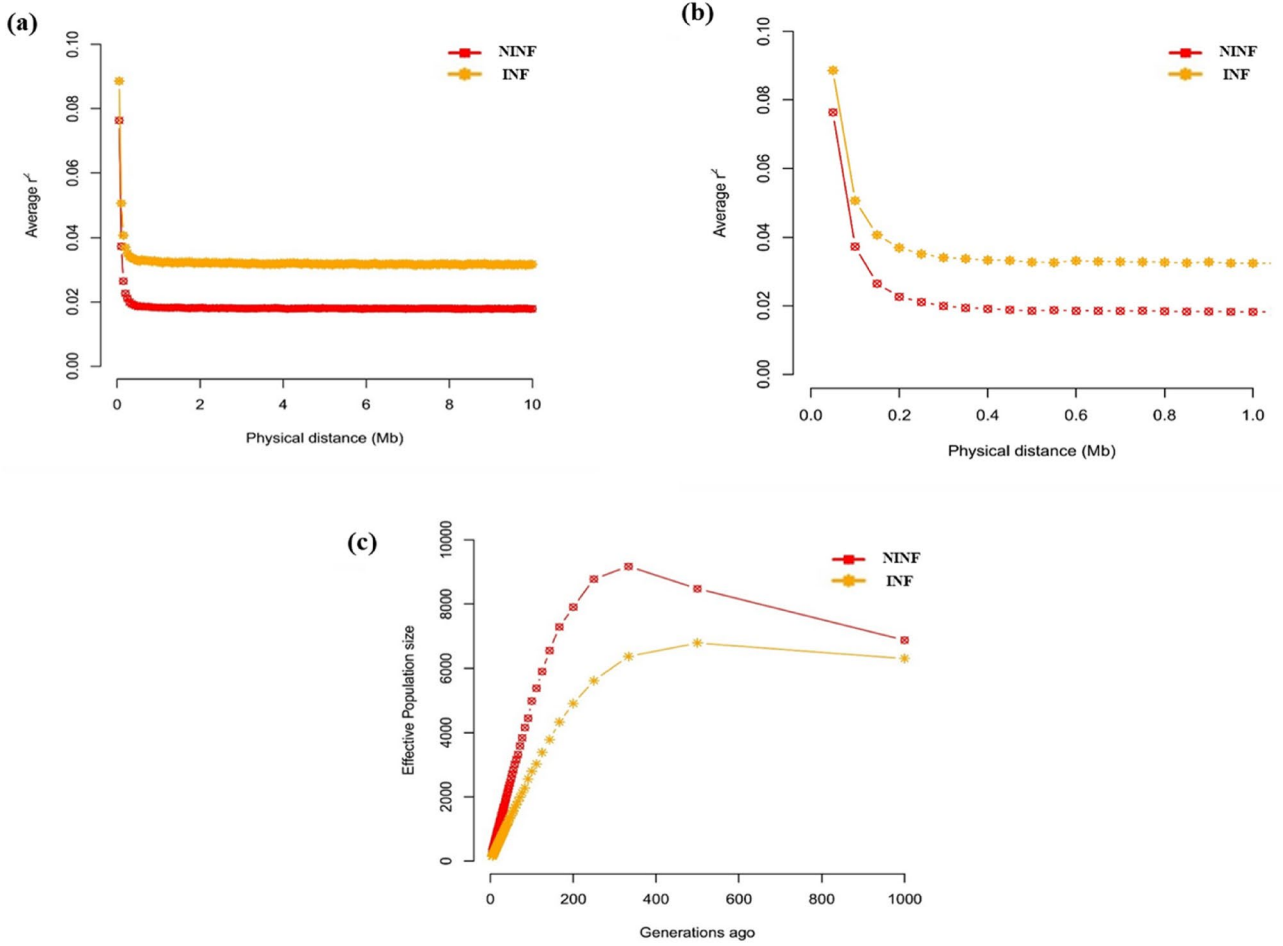
Trends in LD decay (**a**, **b**) and N_E_ across 1000 generations (**c**) in non-infected and infected cohorts of autochthonous Tunisian sheep. *INF* infected cohort; *NINF* non-infected cohort.

The average estimates of *H*_*O*_, *H*_*E*_, *F, F*_*ROH*_ and *ROH* size did not differ significantly (p = 0.05) between the two cohorts (Table 1). However, the non-infected cohort showed marginally higher values of *F* and *ROH* size while the infected animals had marginally higher values of *H*_*O*_, *H*_*E*_ and *F*_ROH_. The number and length of ROH estimated for the 0–5 Mb, 5–10 Mb, > 10 Mb genome length categories are shown in Table 2. The most and least frequent ROH length was observed for the 0–5 Mb and > 10 Mb length categories, respectively. The average length of ROH per animal was highest, in the 5–10 Mb length category for non-infected cohort and > 10 Mb length category for the infected cohort. The two cohorts show similar patterns of LD decay over genomic distance although the non-infected cohort showed overall lower LD (r^2^) (Fig. 1a). In general, the pattern of LD decay shows higher LD at shorter distances which declines rapidly and plateaus off from 0.4 Mb for both cohorts (Fig. 1b). The trend in N_E_ over generation time was the same for both cohorts (Fig. 1c). They are characterised by an increase in N_E_ from 1000 generations ago, which attains maximum value at approximately 330 generations ago, and then declines to the present time. Generally, the non-infected cohort had higher N_E_ across all generations.

**Table 1:** Estimates of genetic diversity parameters for the infected and non-infected cohorts of Tunisian sheep.

Group (sample size)	Observed heterozygosity (*H*_*O*_*)*	Expected heterozygosity (*H*_*E*_)	*ROH* size (Mb)	Inbreeding coefficient (*F*)	*F*_*ROH*_ (genomic inbreeding coefficient)
	(Mean ± Sd)	(Mean ± Sd)	(Mean ± Sd)	(Mean ± Sd)	(Mean ± Sd)
Infected (41)	0.3399 ± 0.0189	0.3522 ± 0.00	3.20 ± 0.7343	0.0349 ± 0.0537	0.0306 ± 0.0514
Non-infected (51)	0.3366 ± 0.0270	0.3500 ± 0.00	3.39 ± 0.6910	0.0381 ± 0.0771	0.0275 ± 0.0646

**Table 2:** Number and length of ROH for each cohort of autochthonous Tunisian sheep for each ROH length category.

ROH statistic	ROH length (Mb)	Infected		Non-infected	
		Mean ± SD	Range	Mean	Range
Number of ROH per animal	0–5	19.44 ± 23.66	2.00–80.00	16.09 ± 26.46	1.00–127.00
	5–10	3.53 ± 7.37	0.00–24.00	3.25 ± 8.80	0.00–46.00
	> 10	0.56 ± 1.27	0.00–4.00	0.56 ± 1.86	0.00–11.00
Length of ROH per animal (Mb)	0–5	1.92 ± 0.47	1.16–2.80	1.83 ± 0.51	1.05–3.07
	5–10	1.81 ± 2.96	0.00–6.97	1.92 ± 2.99	0.00–7.58
	> 10	2.13 ± 4.53	0.00–12.44	1.89 ± 4.46	0.00–15.90

### Population structure analysis

**Figure 2:**
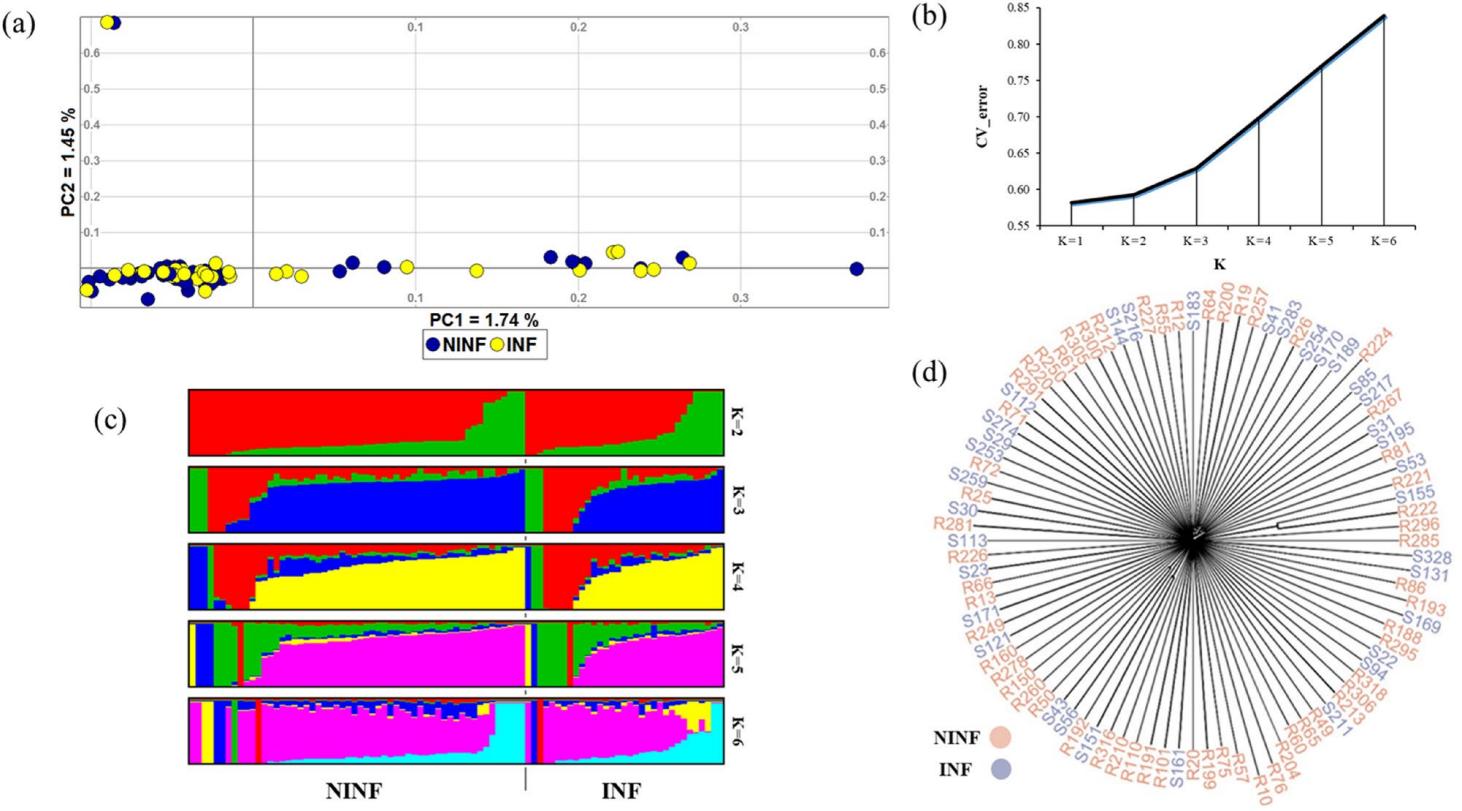
Population genetic structure and phylogenetic analysis of the two cohorts of autochthonous Tunisian sheep (**a**) PCA cluster analysis showing PC1 and PC2; (**b**) Cross-validation plot for admixture analysis; (**c**) Admixture analysis plot showing the genetic backgrounds present in the study cohorts for 2 ≤ K ≤ 6; (**d**) ASD phylogenetic tree of individuals of the study population. *INF* infected cohort; *NINF* non-infected cohort.

Population structure and relationship was investigated using PCA (Fig. 2a), ADMIXTURE tool (Fig. 2b,c) and ASD phylogeny (Fig. 2d). The first and second PC’s of the PCA explained, respectively 1.74% and 1.45% of the total genetic variation. The study animals did not differentiate into distinct genetic groups/clusters that correspond to their infection status. Following ADMIXTURE analysis, the lowest CV error was at *K* = 1 (Fig. 2b) which suggests no genetic differentiation. The ADMIXTURE plot reveals that a similar pattern of genomic composition characterizes the two cohorts at 2 ≤ *K* ≤ 6 (Fig. 2c). This pattern was replicated when ADMIXTURE was performed using three datasets comprising 6000 SNPs each, drawn at random without replacement from the 335,070 SNPs. The ASD phylogeny also showed no genetic stratification (Fig. 2d).

### Genome-wide selection signature analysis

**Figure 3:**
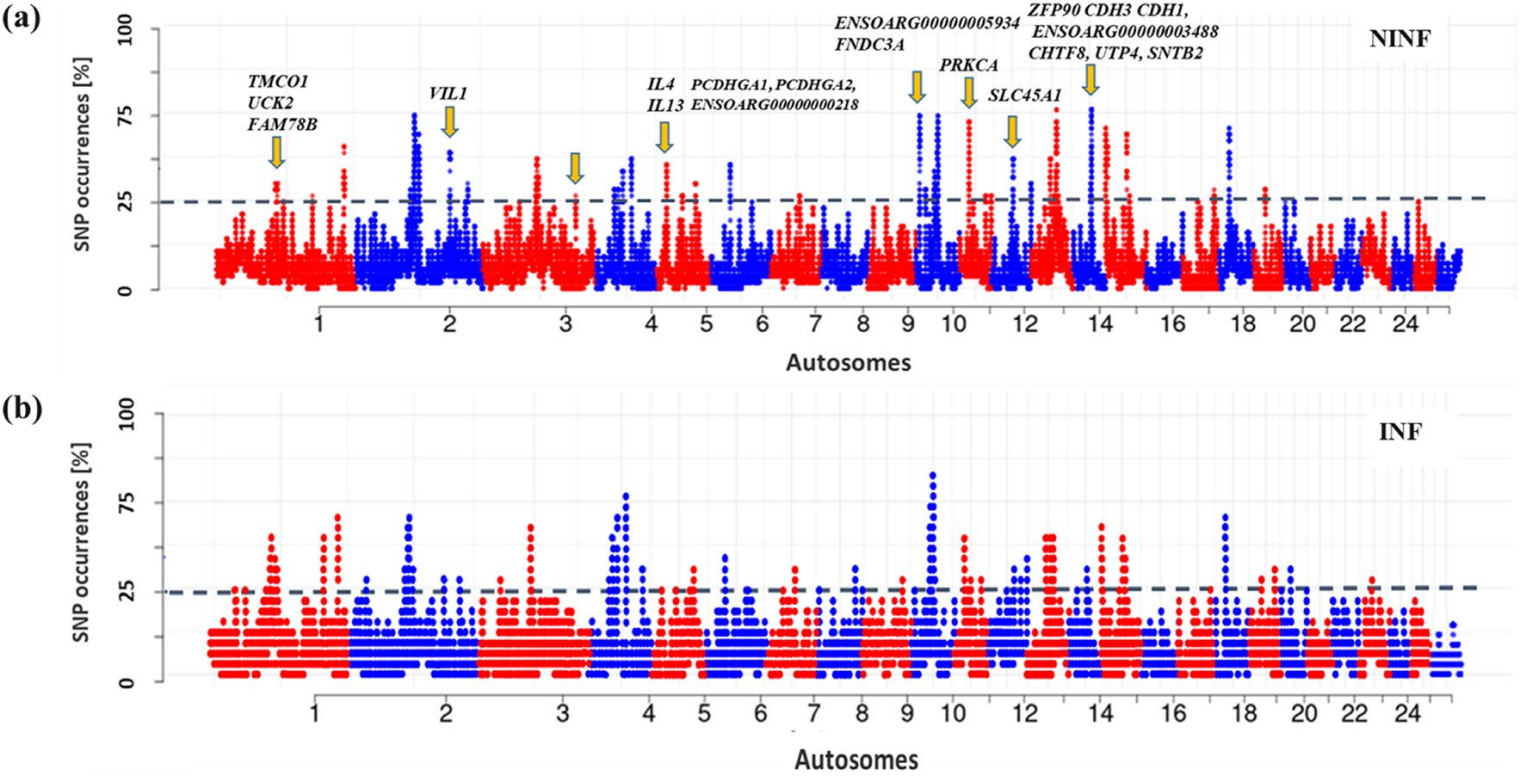
Manhattan plot showing genome-wide distribution frequency of SNPs in stretches of ROH regions. The dashed lines indicate the 25% threshold for each cohort. *INF* infected cohort; *NINF* non-infected cohort.

**Figure 4:**
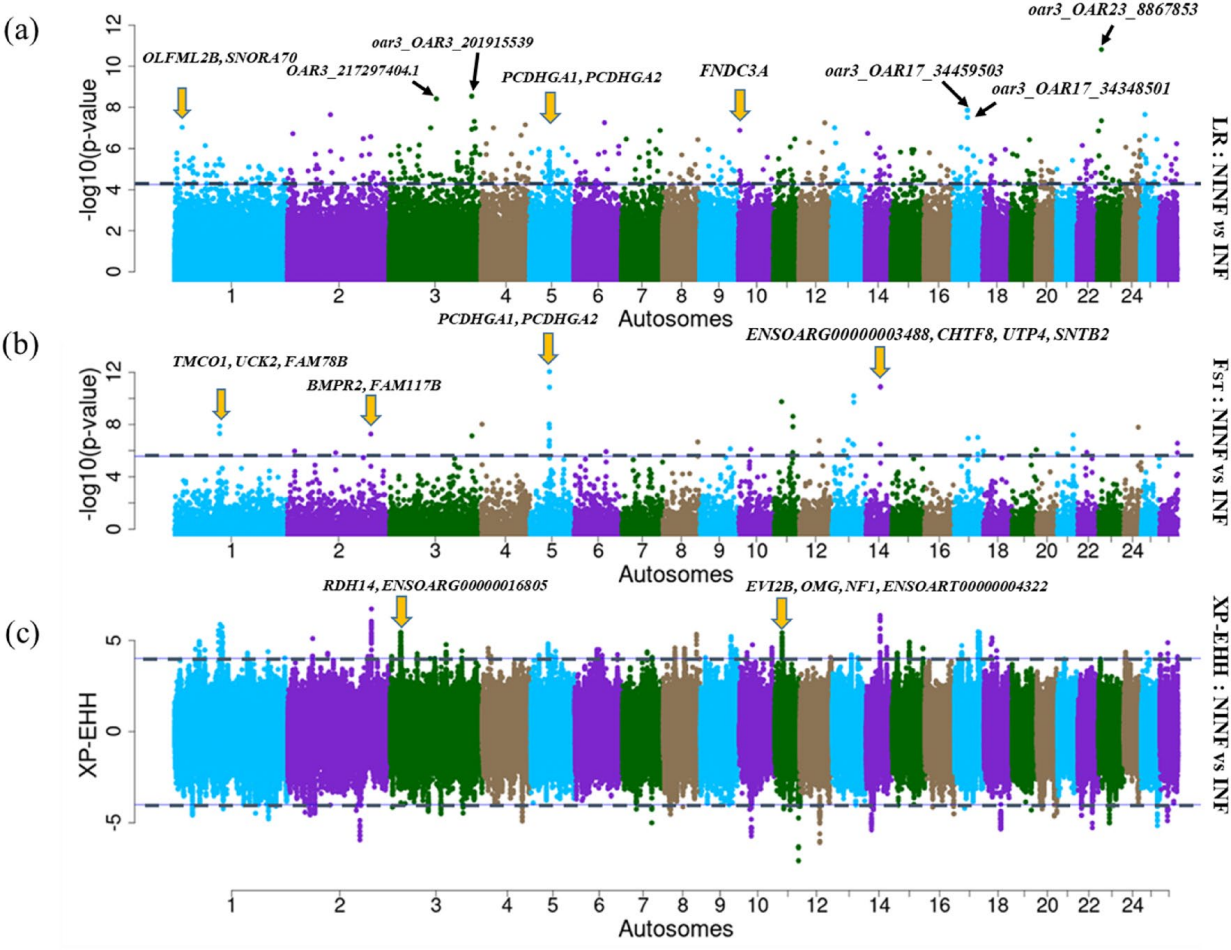
Manhattan plots showing the genome-wide distribution of SNPs following (**a**) LR-GWAS (**b**) F_ST_ and (**c**) XP-EHH analysis using the non-infected and infected cohorts of autochthonous Tunisian sheep. *INF* infected cohort; *NINF* non-infected cohort.

**Figure 5:**
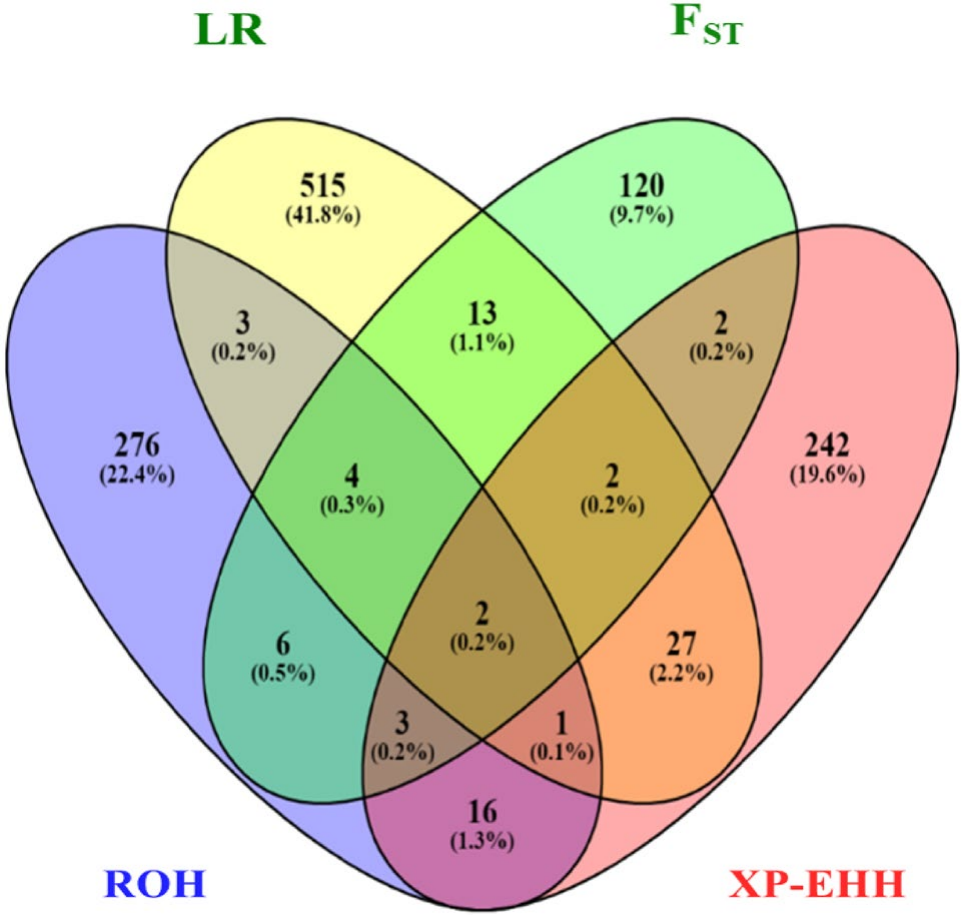
Venn diagram showing the number of genes that were specific and common to the four selection signature methods performed in this study.

The ROH analysis identified 60 ROH regions in the two cohorts (Fig. 3a,b) that spanned 311 genes. The LR-GWAS, F_ST_ and XP-EHH identified 346, 32, and 68 regions (Fig. 4a–c), respectively which spanned 673, 152, and 295 genes. These 446 candidate regions overlapped with 645 genes (Supplementary Table S1) of which 71 were found in candidate regions that were identified by at least two methods (Fig. 5).

We considered the ROH analysis as a method that identifies selection signatures within a cohort. We therefore compared the ROH results of infected and non-infected cohorts (Fig. 3a,b). This identified 23 ROH regions that were specific to the non-infected cohort and which spanned 80 putative genes (Table 3) of which 30 remain uncharacterised/unannotated (prefixed with “ENSOARG”). Six of the 23 candidate regions occurred on OAR 1, 2, 3, 6, 10 and 14 and overlapped with FECGEN, LATRICH_2 and NFEC QTLs (Table 3) that are associated with health traits and in particular, parasite resistance.

**Table 3:** Candidate regions that are specific to the non-infected cohort as identified by ROH analysis.

Region	OAR	Start	Stop	Size (Mb)	No. of SNPs	No. of genes	Genes	QTL, Trait name
1	1	115,934,737	116,254,820	0.320	77	3	TMCO1, UCK2, FAM78B	FECGEN (QTL #13987), Fecal egg count
2	1	188,643,068	188,643,068	0	1	1	FYTTD1	–
3	2	115,482,173	115,484,311	0.002	4	–	–	LATRICH_2 (QTL #12898), Adult and Larva count
4	2	219,142,282	219,594,617	0.452	70	17	RUFY4, CXCR2, CXCR1, ENSOARG00000019495, AAMP, PNKD, TMBIM1, CATIP, SLC11A1, CTDSP1, VIL1, USP37, CNOT9, PLCD4, ZNF142, MIR26B, ENSOARG00000025091	–
5	3	109,370,464	110,015,860	0.645	114	–	–	–
6	3	183,089,260	183,089,260	0.000	1	–	–	NFEC (QTL #17188), Nematodirus FEC
7	4	35,633,277	35,680,007	0.046	9	–	–	–
8	5	18,738,106	19,307,665	0.569	92	23	TMPRSS9, SPPL2B, LSM7, LINGO3, PEAK3, ENSOARG00000025297, DOT1L, SEPTIN8, PLEKHJ1, SF3A2, CCNI2, AMH, AP3D1, ENSOARG00000025298, IZUMO4, MOB3A, KIF3A, IL4, IL13, U6, RAD50, OAZ1, MKNK2	–
9	5	49,789,923	49,930,854	0.140	66	3	LOC101104745 (PCDHGA1), PCDHGA2, ENSOARG00000000218	–
10	5	75,779,568	76,035,485	0.255	55		ENSOARG00000023647	–
11	6	36,519,290	36,522,278	0.002	2	1	ABCG2	FECGEN (QTL #16024), Fecal egg count
12	10	7,142,235	7,746,939	0.604	96	4	ENSOARG00000006632, ENSOARG00000006641, ENSOARG00000026268, ENSOARG00000006647	–
13	10	18,948,900	19,169,123	0.220	46	2	ENSOARG00000005934, FNDC3A	–
14	10	37,089,571	37,098,832	0.009	3	–	–	FECGEN (QTL #13989), Fecal egg count
15	11	61,796,516	61,834,196	0.037	10	1	PRKCA	
16	12	42,716,463	43,245,332	0.528	99	4	ENSOARG00000009688, ENSOARG00000009776, SLC45A1, ENSOARG00000005144	–
17	13	45,726,347	45,826,963	0.100	20	–	–	–
18	13	47,967,869	48,015,537	0.047	8	1	ENSOARG00000026236	–
19	14	35,198,362	35,891,080	0.692	178	15	ZFP90, CDH3, CDH1, TANGO6, PDF, ENSOARG00000003488, CHTF8, UTP4, SNTB2, ENSOARG00000003534, COG8, TMED6, NIP7, TERF2, CYB5B	NFEC (QTL #12893, QTL #12892), Nematodirus FEC
20	15	3,323,204	3,916,206	0.593	112	1	PDGFD	–
21	17	61,855,700	61,872,737	0.017	3	1	LOC101115991	–
22	17	61,944,419	61,970,285	0.025	5	2	ALDH2, ENSOARG00000025686	–
23	17	61,990,418	62,003,285	0.012	3	1	ENSOARG00000025686	–

The LR-GWAS was used to identify candidate regions and possible SNPs associated with GIN infection, while F_ST_, and XP-EHH were also used to investigate selection signatures by contrasting the non-infected and infected cohorts. These three approaches identified 30 candidate regions that overlapped between at least two methods across 17 autosomes (Table 4). When the ROH regions that are specific to the non-infected cohort are considered, the four methods identify 35 candidate regions overlapping between at least two approaches across 19 autosomes and span 121 genes including 11 which remain uncharacterized (Table 4). Of the 35 candidate regions, five that were identified by ROH to be specific to the non-infected cohort overlap with at least one region that was identified by either LR-GWAS, F_ST_ and/or XP-EHH and span 13 genes (Table 4) while one region found on OAR 5 (region number 14) was identified by all the four methods (Table 4). This region spans four genes (LOC101104745, PCDHGA1, PCDHGA2, ENSOARG00000000218) and one QTL trait (BIRTH_WT, Body weight (birth)). None of the four genes are associated, directly or indirectly, with endoparasite resistance. Nineteen regions found across OAR 1, 3, 6, 8, 11, 12, 13, 14, 17, 18 and 21 span FECGEN, TFEC_1, HFEC, NFEC, LATRICH_2, IGA, SAOS, WORMCT, PEPSL and CEOSIN QTLs that are linked to response to GIN infection (Table 4). Ten regions overlapped with production (growth) QTL traits (BW, BIRTH_WT, BONE_WT) across OAR 2, 4, 5 and 6, four regions overlapped with meat and carcass traits associated with fatness QTLs (HCWT, FAT_WT, LMYP) across OAR 2 and OAR 10, and anatomy QTLs (BONE_WT) on OAR 24 (Table 4).

**Table 4:** Candidate regions, associated genes and QTLs that overlapped between at least two methods of detecting selection signatures.

Reg	OAR	Start	Stop	Size (Mb)	Method				Top SNP (LR-GWAS)	P_value*	No. of genes	Genes (Top gene^a^)	QTL, Trait name
1	1	109,500,001	109,800,000	0.300	*–*	*LR*	*F*_*ST*_	–	OAR1_109621789	0.00047	7	CASQ1, PEA15, DCAF8, PEX19, COPA, NCSTN, VANGL2	FECGEN (QTL#13987), Fecal egg count
2	1	111,479,835	111,679,835	0.200	–	*LR*	–	*XP-EHH*	OAR1_111579835	0.00012	3	**OLFML2B**, SNORA70, ENSOARG00000025560	FECGEN (QTL #13987), Fecal egg count
3	1	116,100,001	116,254,820	0.155	*ROH*	*–*	*–*	*XP-EHH*	OAR1_116606247	0.00940	1	**FAM78B**	FECGEN (QTL #13987), Fecal egg count
4	2	60,224,723	60,504,322	0.280	–	*LR*	–	*XP-EHH*	OAR2_60345435	0.00001	1	**PCSK5**	HCWT (QTL#14279), Hot carcass weight; BW (QTL#14280), Body weight slaughter
5	2	203,600,001	203,800,000	0.200	–	*LR*	*F*_*ST*_	*XP-EHH*	OAR2_215596955.1	0.00018	2	**BMPR2**, FAM117B	HCWT (QTL#14279, 14253), Hot carcass weight; BW (QTL# 14280, 14254), Body weight slaughter
6	3	4,000,001	4,300,000	0.300	–	*LR*	–	*XP-EHH*	OAR3_4118423	0.00011	5	AK8, **GTF3C4**, DDX31, AK8, BARX1, CFAP77	HFEC (QTL#12897), Haemonchus contortus FEC
7	3	26,314,565	26,514,565	0.200	–	*LR*	–	*XP-EHH*	OAR3_26114621	0.00001	3	RDH14 (LOC105601856), **LOC101102216**, RDH14 U6	TFEC_1 (QTL #14155), Trichostrongylus colubriformis FEC
8	3	201,773,340	202,015,539	0.242	–	*LR*	*F*_*ST*_	–	OAR3_201915539	0.00006	7	HEBP1, GPRC5D, GPRC5A, **DDX47**, HEBP1, CDKN1B, U6, SEC61B	NFEC (QTL #12882), Nematodirus FEC; FECGEN (QTL #16023), Fecal egg count
9	4	35,495,115	35,695,115	0.200	*ROH*	*LR*	–	–	OAR4_35595115	0.00047	3	**SEMA3D**, U6, ENSOARG00000015210	BW (QTL# 17232), Body weight
10	5	41,700,001	42,000,000	0.300	–	LR	–	XP-EHH	OAR5_41807859	0.00071	9	PDHB, SOWAHA, **SHROOM1**, PDHB, GDF9, UQCRQ, LEAP2, AFF4, ZCCHC10, HSPA4	BIRTH_WT (QTL# 12934), Body weight (birth)
11	*5*	*51,800,001*	*52,200,000*	*0.400*	*–*	*LR*	*–*	*XP-EHH*	OAR5_51959486	0.00015	1	NR3C1	BIRTH_WT (QTL# 12934), Body weight (birth)
12	*5*	*60,100,001*	*60,400,000*	*0.300*	*–*	*LR*	*–*	*XP-EHH*	OAR5_60271682	0.00036	3	SLC2A1, SLC2A2, **FAT2**	BIRTH_WT (QTL# 12934), Body weight (birth)
13	5	19,583,479	19,802,720	0.219	–	*LR*	–	*XP-EHH*	OAR5_19741661	0.00012	4	SLC22A5, SLC22A4, PDLIM4, **P4HA2**	BIRTH_WT (QTL# 12934), Body weight (birth)
14	5	49,789,923	49,930,854	0.141	*ROH*	*LR*	*F*_*ST*_	*XP-EHH*	OAR5_49867927	0.00002	3	LOC101104745 (**PCDHGA1**, (PCDHGA2), ENSOARG00000000218	BIRTH_WT (QTL# 12934), Body weight (birth)
15	*6*	*59,200,001*	*59,500,000*	*0.300*	*–*	*LR*	*–*	*XP-EHH*	OAR6_59306993	0.00031	4	RHOH, U6, **CHRNA9**, RHOH, RBM47	FECGEN (QTL#16024), Fecal egg count
16	6	66,102,303	66,300,000	0.198	*–*	*LR*	*–*	*XP-EHH*	OAR6_66202303		2	**ATP10D**, CORIN	BW (QTL#14284), Body weight (slaughter)
17	7	71,965,934	72,165,934	0.200	*–*	*LR*	*–*	*XP-EHH*	OAR7_72145128	0.00002	3	**KCNH5**, U6, ENSOART00000001032	*–*
18	8	85,300,001	85,600,000	*0.300*	*–*	*LR*	*F*_*ST*_	–	*OAR8_86573428*	0.00019	2	PACRG, **QKI**	LATRICH_2 (QTL #11289912900), Trichostrongylus adult and larva count; FECGEN (QTL# 16025), Fecal egg count
19	10	18,948,900	19,000,000	0.051	*ROH*	–	–	*XP-EHH*	OAR10_18864674	0.00490	1	ENSOARG00000005934	FATWT (QTL#14292), fat weight in carcass; LMYP (QTL #14295), Lean meat yield percentage
20	11	18,200,001	18,400,000	0.200	*–*	*LR*	*–*	*XP-EHH*	OAR11_18201426	0.00001	4	EVI2A, EVI2B, OMG, **NF1**	LATRICH_2 (QTL #12901), Trichostrongylus adult and larva count
21	11	43,600,001	43,900,000	0.300	*–*	*LR*	*F*_*ST*_	–	OAR11_43778741	0.00015	5	ITGA2B, GPATCH8, **FZD2**, CCDC43, DBF4B	LATRICH_2 (QTL #12901), Trichostrongylus adult and larva count
22	11	45,300,001	45,600,000	0.300	*–*	*LR*	*F*_*ST*_	–	OAR11_45447832	0.00029	2	MAPT, **KANSL1**	LATRICH_2 (QTL #12901), Trichostrongylus adult and larva count, IGA (QTL #95626), Immunoglobulin A level
23	12	74,300,001	74,351,694	0.052	*–*	*LR*	*–*	*XP-EHH*	OAR12_74469905	0.00001	1	**CRB1**	IGA (QTL #95627), Immunoglobulin A level
24	13	28,900,001	29,300,000	0.400	–	LR	FST	–	OAR13_29024037	0.00073	2	**FAM171A1**, ITGA8	SAOS (QTL #17198), Salmonella abortusovis susceptibility
25	14	33,865,674	34,065,674	0.200	*–*	*LR*	*–*	*XP-EHH*	OAR14_34068678	0.00021	11	TERB1, NAE1, CA7, PDP2, CDH16, RRAD, CIAO2B (FAM96B), CES2, CES3, CES4A, **CBFB**	NFEC (QTL #12892), Nematodirus FEC; NFEC (QTL #12893), Nematodirus FEC
26	14	35,554,237	35,796,467	0.242	*ROH*	*LR*	*F*_*ST*_	–	OAR14_35677446	0.00050	5	TANGO6, **CHTF8**, UTP4, SNTB2, ENSOARG00000003488	NFEC (QTL #12892)*,* Nematodirus FEC; NFEC (QTL #12893), Nematodirus FEC
27	17	34,359,503	34,611,364	0.252	*–*	*LR*	*F*_*ST*_	–	OAR17_34459503	0.00055	4	**FGF2 (SPATA5)**, NUDT6, U4, ENSOARG00000023095	*–*
28	17	58,100,001	58,578,009	0.478	*–*	*LR*	*F*_*ST*_	*XP-EHH*	OAR17_58269871	0.00071	1	**MED13L**	*–*
29	17	18,417,447	18,500,000	0.083	*–*	*LR*	*–*	*XP-EHH*	OAR17_18517447	0.00017	1	**ENSOARG00000025659**	FECGEN (QTL #16031)*,* Fecal egg count; IGA (QTL #95633), Immunoglobulin A level
30	18	17,337,561	17,537,561	0.200	*–*	*LR*	*–*	*XP-EHH*	OAR18_17468231	0.00001	–	*–*	SAOS (QTL #17199), Salmonella abortusovis susceptibility
31	18	20,100,001	*20,400,000*	*0.300*	*–*	*LR*	*–*	*XP-EHH*	OAR18_20256893	0.00031	9	TICRR, KIF7, PLIN1, PEX11A, **WDR93**, TRNAK-CUU, MESP2, ANPEP	WORMCT (QTL#19806), Worm count; SAOS (QTL #17199), Salmonella abortusovis susceptibility
32	21	38,800,001	39,100,000	0.300	*–*	*LR*	*–*	*XP-EHH*	OAR21_38935734	0.00010	1	**PAG6**	PEPSL (QTL#126104, 126105*, *126106) Pepsinogen level; CEOSIN (QTL#14157), Change in eosinophil number; SAOS (QTL #17195), Salmonella abortusovis susceptibility
33	24	3,724,161	3,927,939	0.204	*–*	*LR*	*–*	*XP-EHH*	OAR24_3802371	0.00001	9	C16orf96, **MGRN1**, NUDT16L1, ANKS3, SEPTIN12 (SEPT12), ROGDI, GLYR1, C24H16orf71, ENSOARG00000003885	BONE_WT (QTL#14315), Bone weight in carcass
34	25	14,000,001	14,300,000	0.300	–	LR	–	XP-EHH	OAR25_14136891	0.00091	1	**BICC1**	–
35	26	42,900,001	42,931,347	0.031	*–*	*LR*	*F*_*ST*_	*XP-EHH*	OAR26_43044064	0.00002	1	**ZNF385D**	*–*

*Significant markers following LR-GWAS Bonferroni correction P < 0.001

^a^Top genes found close to the top-most significant SNP marker are shown in bold.

Functional enrichment analysis was first tested in the pool of 51 genes, excluding uncharacterized genes, that are present in the 23 ROH candidate regions that were specific to the non-infected cohort (Table 3). A second functional enrichment analysis was performed with the set of 110 genes, excluding 11 uncharacterized ones that were present in the 35 candidate regions that overlapped between at least one ROH, LR-GWAS, F_ST_ and XP-EHH regions (Table 4). We found two functional term clusters that were significantly (enrichment score > 1.5) enriched for the genes present in the ROH regions of the non-infected cohort (Table 5). These were associated with “immune system process” and “cytokine receptor interaction”. The second analysis resulted in three significantly (enrichment score > 1.5) enriched clusters. The first cluster was associated with the GO biological terms “Carboxylesterase type B” and “Hydrolase activity”. The “Apical plasma membrane” term, which has a role in intestinal innate immunity, and “Integrin signalling” that functions in immune cells were among the most significant GO biological terms in the second and third clusters, respectively.

**Table 5:** Enriched functional term clusters following *DAVID* analysis of genes identified by ROH analysis in candidate regions that were specific to the non-infected cohort and those that overlapped between at least two methods of detecting selection signatures.

	Cluster	Score	Category	Term	Gene count	P-value	Genes	Benjamini
ROH regions specific to non-infected cohorts = 51 genes	1	2.19	INTERPRO	IPR001325:Interleukin-4/interleukin-13	3	0.00003	IL4, IL13	0.002
			INTERPRO	IPR018096:Interleukin-4/interleukin-13, conserved site	3	0.00003	IL4, IL13	0.002
			SMART	SM00190:IL4_13	3	0.00004	IL4, IL13	0.002
			KEGG	oas04060:Cytokine-cytokine receptor interaction	6	0.00056	IL4, CXCR1, CXCR2, IL13, AMH	0.057
			KEGG	oas05310:Asthma	3	0.00638	IL4, IL13	0.326
			INTERPRO	IPR012351:Four-helical cytokine, core	3	0.01402	IL4, IL13	0.364
			INTERPRO	IPR009079:Four-helical cytokine-like, core	3	0.01551	IL4, IL13	0.364
			KEGG	oas04664:Fc epsilon RI signaling pathway	3	0.01706	IL4, IL13	0.580
			KEGG	oas05321:Inflammatory bowel disease (IBD)	3	0.02290	IL4, IL13	0.584
			KEGG	oas05162:Measles	3	0.06509	IL4, IL13	1.000
			KEGG	oas04630:Jak-STAT signaling pathway	3	0.09399	IL4, IL13	1.000
			UP_KEYWORDS	Signal	8	0.52067	IL4, PDF, CDH1, IL13, AMH, TMED6, TMCO1	1.000
			GOTERM_CC	GO:0005615 extracellular space	3	0.67367	IL4, IL13	1.000
	2	1.73	KEGG	oas04060:Cytokine-cytokine receptor interaction	6	0.00056	IL4, CXCR1, CXCR2, IL13, AMH	0.057
			UP_KEYWORDS	Growth factor	4	0.00161	IL4, PDGFD, AMH	0.074
			UP_KEYWORDS	Secreted	3	0.32286	IL4, AMH	1.000
			UP_KEYWORDS	Disulfide bond	5	0.39333	IL4, TMPRSS9, LOC101115991, AMH	1.000
Overlapping genes = 56 genes (excluding ENSOARG genes	1	3.00	INTERPRO	IPR019826: Carboxylesterase type B, active site	3	0.00022	CES3, CES4A, CES2	0.028
			INTERPRO	IPR002018:Carboxylesterase, type B	3	0.00070	CES3, CES4A, CES2	0.045
			GOTERM_MF	GO:0016787 hydrolase activity	4	0.00118	CES3, NUDT6, CES4A, CES2	0.051
			UP_KEYWORDS	Hydrolase	7	0.00520	DDX47, ATP10D, CES3, PCSK5, NUDT6, CES4A, CES2	0.192

## Discussion

GIN infections and associated gastroenteritis impact negatively the production efficiency of ruminant livestock, and their management is essential to meet future demand for animal source foods. The observation of large variations in GIN infection suggests variability at the genome level that underpins inter-animal variability in susceptibility^39, 40^. Rouatbi et al.^23^ observed inter-individual variation in GIN infection in autochthonous Tunisian sheep leading to the suggestion that it could have a genetic basis. We therefore generated and analysed Ovine 600 K SNP BeadChip genotype data, from 92 samples that comprised GIN infected and uninfected animals under field challenge from Rouatbi et al.^23^ study. The aim was to investigate signatures of variability in GIN infection and resistance in autochthonous Tunisian sheep that are managed under natural grazing and no history of anthelmintic intervention. We applied four methods to detect genomic regions associated with GIN resistance. Although our study yielded some interesting findings, we acknowledge that studies that use naturally acquired field exposure to deliver a challenge always run the risk of uninfected animals being a combination of truly highly resistant animals, animals that never saw infection and animals that have had infection cleared due to chemical treatment. Additionally, producer provided information is at times not always accurate. These factors may serve to dilute the certainty that the two groups are in fact functionally dissimilar.

Although we expected the two cohorts to show differences in genetic diversity and structure, this was not the case; they showed similar levels of genetic diversity and inbreeding and the three clustering algorithms provided corroborating evidence of lack of stratification that was consistent with infection status. The lack of genetic differentiation and structure was also reported between prolific and non-prolific cohorts of Bonga sheep from Ethiopia^41^ and in the Brazilian Santa Inês breed which shows variability in resistance to GIN infection^19^. The absence of genetic stratification appears to be a characteristic of sheep from the Maghreb as it has also been observed in sheep populations from Algeria^42^ and Morocco^43^ implicating extensive intermixing and cross-mating. There are four possible explanations for the lack of genomic stratification corresponding to infection status. (1) The selection pressure driving the differences in GIN infection is/has not been stringent or long enough to result in differentiation at the genome level; (2) the level of parasite infection may be too low to result in meaningful genomic variation; (3) it may point to a lack of farmer-driven selection that is biased towards the use of uninfected animals for breeding; and/or (4) there could be a high natural selection pressure whereby all animals effectively have the resistance genotype and therefore rather than a distribution, spanning susceptible to resistant, there is a skewed distribution that spans “moderately resistant” to “highly resistant” individuals. One or all of these reasons may explain the lack of genetic differentiation between the two cohorts. The level of genetic diversity in the two cohorts is higher than that observed in commercial breeds, but it falls within the range of values reported in other sheep found in Africa and China^44–46^.

We assessed the average length and number of ROH per individual at three genomic distance categories and the trends in N_E_ over generation time for each cohort. The two cohorts had a high frequency of ROH in the shorter (0–5 Mb) length category reflecting older evolutionary events such as past or ancestral inbreeding. The two cohorts showed similar patterns and trends in LD decay and N_E_. However, the non-infected cohort had higher values of N_E_ across all generations. This difference is difficult to explain but we presume it may be the result of higher mortalities in the infected cohort. Both cohorts show a gradual increase in N_E_ from 1000 generations ago. This is followed by a drastic decline from around 330 generations ago to the present time suggesting a bottleneck event. A similar trend was observed in Ethiopian and Sudanese local sheep^47^. Assuming a generation time of three years for traditionally managed local sheep, it can be inferred that the increase in N_E_ started around 3000 years ago. The start of the decline in N_E_ 330 generations back translates to around 990 years ago. Between 240 and 1140 years ago, three favourable climatic periods interspersed with short extreme dry spells were experienced in the continent^48^. We suggest that the sheep populations most likely thrived when conditions were optimal but shrunk during subsequent droughts. The footprints of these demographic perturbations appear to have been retained in the genomes of the indigenous sheep.

Our analysis revealed 35 candidate regions, spanning 110 genes, that overlapped between at least two out of the four methods we used to detect genomic regions associated with differences in endo-parasite infections. The identification of overlapping candidate regions that are under selection by different approaches suggests plausible evidence for selective influences at the genome^49^. The convergence of our results points to the reliability of our findings and suggest that they are unlikely to be the outcome of chance events or analytical artefacts.

Both the within (ROH) and between-population (LR-GWAS, F_ST_, XP-EHH) approaches identified genomic regions that could be driving GIN resistance in autochthonous Tunisian sheep. At least two or more methods identified the same candidate regions that colocalized with the FECGEN, TFEC_1, HFEC, NFEC, LATRICH_2, IGA, SAOS, WORMCT, PEPSL and CEOSIN QTLs that have been associated with health traits and in particular parasite resistance, immune capacity and disease susceptibility in sheep showing resistance to GIN^50–52^. This result indicates that some common QTLs underly parasite resistance traits in sheep and support a role for convergent evolution in driving host GIN resistance. For instance, the FECGEN QTL which results in reduced FEC in unmanaged, naturally-parasitized domestic sheep^53^ and in the primitive Soay sheep^20^, has been associated with a microsatellite allele (o(IFN)-γ_126_) found in the first intron of the interferon gamma (IFN)-γ gene and with increased titre of *Teladorsagia circumcincta*-specific IgA. It has been shown that the effects of the o(IFN)-γ_126_ allele and IgA on FEC are not independent, and that IgA may mediate the (IFN)-γ effect on FEC^20^. Furthermore, the IGA QTL could be related to early response to incoming larvae, whereas the FECGEN QTL may be associated with the ability to avoid the development of adult parasites^52, 54^. The fact that the candidate regions spanned QTLs associated with different QTL traits was expected because the animals analysed in this study are grazed in communal pastures where challenge from multiple GIN parasite species is common and could present different aspects of host-parasite interaction during infection. Indeed, eight nematode species, *T. circumcincta*, *T. trifurcate*, *Haemonchus contortus*, *Marshallagia marshalli*, *Trichostrongylus vitrines*, *T. axei*, *Ostertagia lyrate*, *O. ostertagi* and *O. occidentalis*, were found to colonize the abomasum of the study individuals^23^.

QTL and GWAS studies suggest that GIN infections can evoke several host responses that enhance innate and acquired immune responses, gastric mucosal protection, haemostasis pathways, delay in parasite development and reduction in number of eggs produced by GIN^16^. These manifestations depend on the nematode species, parasite exposure, and host-specific factors including age, sex, genetic make-up, hormonal and nutritional status^55^. The animals analysed in our study are grazed under natural open pastures and are exposed to a wide range of GIN. Our expectation, therefore, was that they would exhibit a large repertoire of host defence mechanisms which may be reflected in the functions of the putative genes present in the candidate regions. We observed two organic cation transporters, SLC22A4 (OCTN1) and SLC22A5 (OCTN2) which are carriers of hydroxyurea, in an overlapping candidate region on OAR 5 (Table 4). Hydroxyurea can inhibit ribonucleotide reductase (RR) and is retained at high concentrations in tissues of various mammalian species including sheep^56^. Compared to viral or bacterial ribonucleotide reductase, the mammalian RR is less susceptible to inhibition by hydroxyurea^56^. This action can be critical in inhibiting viral and bacterial replication without affecting mammalian cellular growth. Hydroxyurea can therefore offer a level of immunity against GIN infection as part of the innate immune defence system which may inhibit endoparasites in the gastrointestinal tract of non-infected animals. Some variants present in SLC22A4 and SLC22A5 have also been associated with Inflammatory Bowel and Crohn’s disease’s in humans^57, 58^.

We observed BMPR2 (Bone Morphogenetic Protein Receptor Type 2) in an overlapping candidate region on OAR 2 (Table 4). Ligands of this gene are members of the TGFβ superfamily whose homologues are key players in inducing immunological tolerance^59^. Elevated expression of TGFβ have been observed in mammalian hosts that mount an effective immune response against GIN^59–62^. BMPR2 was found to be highly expressed in mesenteric lymph nodes of cattle that were selected for resistance to intestinal nematodes^63^ suggesting association with parasite resistance. BMPR2 also occurred in a cluster of genes that were upregulated following *Eimeria acervulina* infection in chicken and associated with the functional term “Inflammatory Response” in the “Disease and Disorders” category^64^. Several mechanisms may operate to increase the levels of TGFβ under parasite infection including, host homeostasis to minimize immunopathology under chronic infection; pathogens triggering TGFβ production or activation; or parasite mimicry of the host cytokine to drive the same pathway as the hosts TGFβ^59^. Examples of all three have been reported in diverse sheep breeds^59^ and the one that operates in autochthonous Tunisian sheep remains unknown calling for further investigation.

The IL-4 (Interleukin-4) and IL-13 (Interleukin-13) occurred in a candidate region on OAR 5 that was specific to the non-infected cohort. IL-4 plays a crucial role in the differentiation of naive T helper (Th) cells into Th2 effector cells which promote humoral immunity and provide protection against intestinal helminths^65^. In sheep, Jacobs et al.^66^ observed that impaired IL-4 signalling promoted the establishment of *H. contortus* and increased larval burden. An increase in IL-13 in intestinal lymph cells was observed in sheep selected for increased resistance to nematodes^67^. Two genes RUFY4 (RUN and FYVE Domain Containing Protein 4) and VIL1 (Villin 1) and two IL-8 receptors (CXCR1 and CXCR2) occurred in a strong candidate region on OAR 2 that was also identified by ROH to be specific to the non-infected cohort. RUFY4 is a positive regulator of autophagy and is expressed in a cell-specific manner or under specific immunological conditions associated with IL-4 expression^68^. VIL1, has been shown to protect gut epithelial cells by decreasing epithelial damage^69^. It was among the top 30 proteins that were differentially regulated between resistant and susceptible sheep and may play an important role in maintaining the epithelial integrity of abomasal mucosa in response to haemonchosis^70^. It can therefore be hypothesized that the interaction between RUFY4 and VIL1 with IL-4 may enhance long-term endoparasite resistance in sheep. The two IL-8 receptors were found to be expressed and distributed in normal and morphologically damaged large intestines, suggesting that IL‐8 may play an important role in mediating immune response in gastrointestinal tract, beyond that of potentiating neutrophil recruitment and inflammation^71^ following intestinal epithelial damage by endoparasites.

Protective immunity against GIN and subsequent parasite expulsion is known to be mediated by Th2 immune response that is orchestrated by the secretion of cytokines such as IL-4, IL-5, IL-10 and IL-13, which promote the recruitment of eosinophils, basophils, mast cells, goblet cell hyperplasia and concurrent mucus and antibody production^72–74^. Nematode expulsion is known to rely on smooth muscle contraction and increased mucus production^75^. The latter was found to be mediated by intelectin 1 (ITLN), a calcium dependent lectin, that is expressed by Paneth cells in the small intestine of mouse^76^ and mucus neck cells in the abomasum of sheep^77^. The expression of ITLN in sheep goblet cells was found to be upregulated by IL-4^77^. High expression of ITLN was also found to be induced during nematode expulsion in *Trichinella spiralis* and *Nocardia brasiliensis* in rodents^78, 79^ and *T. circumcincta* infections in sheep^77, 80^. Th2 response has also been described as a mediator for acute wound healing during helminth infection^81^. We therefore suggest that the upregulation of ITLN by IL-4 may play a role in enhancing GIN resistance in autochthonous Tunisian sheep through parasite expulsion and enhancing wound healing.

Other possible candidate genes that have been associated with GIN resistance and occur in our overlapping candidate regions include FGF2*,* FAM78B*,* SPATA5, SPPL2B and FAM96B. FGF2 also known as basic fibroblast growth factor (bFGF and/or FGF-β), is a growth factor and signaling protein that can synergize with IL-17 in the gut to activate the ERK pathway and induce genes for repairing damaged intestinal epithelium^82^. The gut epithelium is essentially the first line of defence against microbiota and pathogens and therefore, it plays a critical role in enhancing mucosal immunity. FAM78B (Family with Sequence Similarity 78-Member B) was in an overlapping region on OAR 1. In a genome-wide association study of endo-parasite phenotypes, FAM78B was found in a region on bovine chromosome 3 that spanned SNPs that were most strongly associated with antibody response to *O*. *ostertagi*^40^. SPATA5 (Spermatogenesis Associated 5) was among five genes that were included in the most significant functional term cluster linked with immunity-related and cell-proliferation processes in an investigation of genomic regions and genes for gastrointestinal parasite resistance in Djallonke sheep^83^. SPPL2B (Signal peptide peptidase-like 2B), a member of the signal peptide peptidase-like protease (SPPL) family, localizes to endosomes, lysosomes and plasma membrane and plays a role in enhancing innate and adaptive immunity by cleaving TNFα in activated dendritic cells that triggers IL-12 production^84^. In humans and chicken, there is strong evidence linking polymorphisms in FAM96B (family with sequence similarity 96 member B) with gastrointestinal and metabolic diseases, and with the development of the digestive system and disorder networks^85, 86^. These findings suggest that several mechanisms may be employed to trigger and sustain GIN resistance in sheep under traditional grazing and exposure to multiple GIN species.

Our analysis revealed several candidate genomic regions spanning a number of production (growth), and meat and carcass (fatness and anatomy) QTLs. This result was unexpected given our analytical strategy. Kipper et al.^87^ reported a reduction of 5% and 31% in average daily feed intake and average daily weight gain, respectively in parasitized pigs. Endoparasites tend to limit host nutrient availability by reducing host food intake, digestion, absorption and nutrient assimilation, resulting in nutritional deprivation and destabilization of host growth and development^88, 89^. To counter against these negative effects, we suggest that natural selection may be acting on the regions spanning growth QTLs to ensure growth and developmental stability of the study populations under GIN challenge. The parallel selection signatures overlapping the growth QTLs may therefore be adaptive strategy that ensures the survival of the study populations.

In conclusion, we assessed the diversity and population structure of two cohorts (infected and non-infected) of autochthonous sheep from Tunisia that were classified based on FEC levels. The two cohorts were characterised by similar levels of genetic diversity and the same genome background suggesting common history and genetic admixture. Four methods of detecting selection signatures identified regions of the genome that were most likely associated with GIN resistance suggesting that the animals have established a certain level of immunity under natural challenge. The functions of the putative genes and the overlapped QTLs suggest multiple strategies, including host immune response, intestinal epithelium damage repair, mucus production and parasite expulsion, play a role in GIN resistance in sheep. This may be due in part to the fact that GIN resistance is the net outcome of many physiological and immunological pathways, and thus resistance in animals could be owing to variation at a large number of loci. Furthermore, natural selection is also acting concurrently on regions spanning growth related QTLs to ensure developmental stability under GIN challenge. Although the data used in our study is relatively small, we believe this does not compromise the integrity of our findings as it is compensated for by the high density of the marker loci used. We have also limited our discussion to candidate regions that were specific to the non-infected cohort and those that overlap between at least two analytical approaches. This research confirms the importance of obtaining data from local sheep populations managed in their production environments to gather information on genomic regions of functional significance in GIN resistance.

## Supplementary Information

Supplementary Information.

## Data Availability

The data used in here is available from the communicating author upon reasonable request.
