# Supplementary material for: Genome-wide insights on gastrointestinal nematode resistance in autochthonous Tunisian sheep
Source: Sci Rep. 2021 Apr 29;11:9250. doi: 10.1038/s41598-021-88501-3 (PMC8085236; doi:10.1038/s41598-021-88501-3)
Supplement: Supplementary file 1 [file 41598_2021_88501_MOESM1_ESM.odt]

Supplementary Information

Genome-wide insights on gastrointestinal nematode resistance in autochthonous Tunisian sheep

Ahbara AM1,2, Rouatbi M3, Gharbi, M3, Rekik M1, Haile A1, Rischkowsky B1, Mwacharo JM1,4,5*

1Small Ruminant Genomics, International Centre for Agricultural Research in the Dry Areas (ICARDA), Addis Ababa, Ethiopia

2Department of Zoology, Faculty of Sciences, Misurata University, Misurata, Libya

3Laboratory of Infectious Animal Diseases, Zoonosis and Sanitary Regulation, University of Manouba, and Institution of Agricultural Research and Higher Education, National School of Veterinary Medicine of Sidi Thabet, Tunisia.

4Animal and Veterinary Sciences, Scotland’s Rural College (SRUC) and Centre for Tropical Livestock Genetics and Health (CTLGH), The Roslin Institute, Midlothian, United Kingdom

*Communicating author: [j.mwacharo@cgiar.org](mailto:j.mwacharo@cgiar.org)

Supplementary Table S1. Enriched functional term clusters and their enrichment scores following DAVID analysis for genes identified to be associated with resistance to internal parasite in Tunisian sheep.

Supplementary Table S1. Enriched functional term clusters and their enrichment scores following DAVID analysis for genes identified to be associated with resistance to internal parasite in Tunisian sheep.

| No. | Term | Function | No. of genes | P-value | Genes |
| --- | --- | --- | --- | --- | --- |
| 1 | ssc04060 | Cytokine-cytokine receptor interaction | 19 | 0.00007 | IL10, CXCL9, IL25, TNFRSF12A, IL15, IFNGR1, IL1R2, IL5RA, PPBP, CXCL13, CXCL10, CXCL11, IL18RAP, IFNG, CLCF1, IL2RB, RELT, IL18R1, IL17A |
| 2 | GO:0006954 | Inflammatory response | 18 | 0.00008 | IL10, CXCL9, IL25, PPBP, CXCL13, LTB4R, TLR1, CXCL10, HRH1, CXCL11, KIT, MAPKAPK2, C3AR1, TLR10, IL17F, TLR6, RELT, IL17A |
| 3 | ssc05321 | Inflammatory bowel disease (IBD) | 10 | 0.00012 | SMAD2, IL10, IL18RAP, SMAD3, IFNG, IFNGR1, IL17F, NFKB1, IL18R1, IL17A |
| 4 | GO:0004089 | carbonate dehydratase activity | 6 | 0.00014 | CA12, CA1, CA3, CA4, CA7, CA14 |
| 5 | IPR023561 | Carbonic anhydrase, alpha-class | 6 | 0.00021 | CA12, CA1, CA3, CA4, CA7, CA14 |
| 6 | IPR002957 | Keratin, type I | 8 | 0.00026 | K38, KRT15, KRT35, KRT34, K33, V15, KRT32, KRT9 |
| 7 | PIRSF001191 | matrix metalloproteinase, stromelysin type | 6 | 0.00030 | MMP12, MMP27, MMP1, MMP3, MMP8, MMP20 |
| 8 | oas00910 | Nitrogen metabolism | 6 | 0.00032 | CA12, CA1, CA3, CA4, CA7, CA14 |
| 9 | IPR001148 | Alpha carbonic anhydrase | 6 | 0.00033 | CA12, CA1, CA3, CA4, CA7, CA14 |
| 10 | GO:0030496 | midbody | 14 | 0.00033 | SDCCAG3, PLK1, HEPACAM2, GNAI3, ANXA11, IQGAP1, VPS37B, HSP90B1, PRC1, ZFYVE19, CHMP4B, KIF20A, RAB11FIP4, SPG20 |
| 11 | GO:0070527 | Platelet aggregation | 6 | 0.00039 | P2RY12, RAP2B, ITGB3, PLEK, MYH9, VCL |
| 12 | SM01057 | SM01057 | 6 | 0.00048 | CA12, CA1, CA3, CA4, CA7, CA14 |
| 13 | IPR016293 | Peptidase M10A, metazoans | 6 | 0.00049 | MMP12, MMP27, MMP1, MMP3, MMP8, MMP20 |
| 14 | GO:0002690 | Positive regulation of leukocyte chemotaxis | 5 | 0.00054 | CXCL10, CXCL9, CXCL11, PPBP, CXCL13 |
| 15 | GO:0042742 | Defense response to bacterium | 9 | 0.00074 | IL10, ANKRD17, TLR1, FCER1G, IFNG, CEBPE, TLR6, CXCL13, HAMP |
| 16 | GO:0048535 | Lymph node development | 5 | 0.00098 | RIPK3, IL15, FADD, CXCL13, NKX2-3 |
| 17 | GO:0009897 | External side of plasma membrane | 12 | 0.00100 | CD79B, CXCL10, IL1RL1, CXCL9, ACE, FCER1G, IFNG, ITGB3, KIT, FCER1A, CD34, IL17A |
| 18 | IPR001818 | Peptidase M10, metallopeptidase | 6 | 0.00176 | MMP12, MMP27, MMP1, MMP3, MMP8, MMP20 |
| 19 | IPR018487 | Hemopexin-like repeats | 6 | 0.00229 | MMP12, MMP27, MMP1, MMP3, MMP8, MMP20 |
| 20 | GO:0004715 | non-membrane spanning protein tyrosine kinase activity | 8 | 0.00230 | WEE1, TEC, SRMS, PEAK1, FES, TXK, DYRK1A, PTK6 |
| 21 | GO:0008270 | zinc ion binding | 65 | 0.00377 | ZCCHC10, OTUD7B, TTF2, NR3C1, MMP20, CA1, RNF114, MMP27, CA3, ZMIZ1, ANPEP, TRIM2, CA4, CA7, ARIH2, ZNF385D, RAG2, LMCD1, EARS2, RAG1, ZCCHC24, LIMCH1, APOBEC4, MMP1, USP3, MMP3, MMP8, MMP12, TRAF6, S100A5, TRIM59, MATR3, UTRN, RAPSN, S100A8, PAPPA2, KMT2D, KMT2B, ZDHHC23, DTX2, RNF2, ADAMTS14, ABLIM1, ZDHHC19, MAN2A2, MICAL1, UBR5, ZNF622, PDLIM4, RNF133, TRIM45, CA12, KDM4A, PHC2, ZFHX3, SEC24A, DTNA, NFXL1, PIKFYVE, RNF148, RNF149, AGBL2, NEURL1, RNF182, TRIM38 |
| 22 | GO:0001656 | metanephros development | 5 | 0.00519 | APH1A, EYA1, NF1, ITGA8, HOXC11 |
| 23 | GO:0001578 | microtubule bundle formation | 6 | 0.00595 | PRC1, MAP2, FES, PLK1, KIF20A, CLASP1 |
| 24 | IPR006026 | Peptidase, metallopeptidase | 6 | 0.00675 | MMP12, MMP27, MMP1, MMP3, MMP8, MMP20 |
| 25 | IPR018338 | Carbonic anhydrase, alpha-class, conserved site | 4 | 0.00909 | CA12, CA1, CA3, CA7 |
| 26 | GO:0005794 | Golgi apparatus | 36 | 0.01097 | TRRAP, GLIS3, GNAI3, HEPACAM2, PCSK5, APH1A, NCSTN, LAPTM4A, DUSP10, CUX1, CAPN2, NAA25, RHO, CTNNA1, ZNF622, CLASP1, FKTN, ENPP7, SLC30A7, PLA2G12A, KCNJ6, SYT1, DDX31, AP1AR, TERF2, EXT2, SLC9A8, KCNS3, ERF, TAF7, FAM20C, KIF20A, ZP3, ATF6, RAPSN, GLYR1 |
| 27 | oas04330 | Notch signaling pathway | 7 | 0.01326 | DLL4, APH1A, PSENEN, NCSTN, APH1B, DTX2, MAML3 |
| 28 | GO:0005882 | intermediate filament | 8 | 0.01400 | K38, KRT15, KRT35, KRT34, K33, V15, KRT32, KRT9 |
| 29 | IPR019466 | Matrilin, coiled-coil trimerisation domain | 3 | 0.01487 | MATN1, MATN3, MATN2 |
| 30 | GO:0009952 | anterior/posterior pattern specification | 10 | 0.01580 | VANGL2, CDX2, HOXC5, HOXC4, HOXC9, PCSK5, HOXC8, HOXC11, PBX1, HOXC10 |
| 31 | GO:0030901 | midbrain development | 5 | 0.02137 | BARHL1, TAL2, RFX4, WLS, PITX3 |
| 32 | GO:0006730 | one-carbon metabolic process | 5 | 0.02137 | CA12, AHCYL2, CA1, CA3, CA7 |
| 33 | IPR010600 | Inter-alpha-trypsin inhibitor heavy chain, C-terminal | 3 | 0.02394 | ITIH4, ITIH3, ITIH1 |
| 34 | IPR003599 | Immunoglobulin subtype | 26 | 0.02410 | CD83, FLT3, AMIGO1, HEPACAM2, VTCN1, FCAR, MYOM2, PTPRF, CD19, SLAMF7, NCAM1, PTGFRN, VSTM5, CD96, KIRREL2, DCC, FCRL3, FCRL4, UNC5C, GP6, NCR1, LRFN3, NPHS1, LRIT3, CD101, CD244 |
| 35 | GO:0015193 | L-proline transmembrane transporter activity | 3 | 0.02461 | SLC36A1, SLC36A2, SLC36A4 |
| 36 | GO:0032922 | circadian regulation of gene expression | 7 | 0.02608 | PPP1CB, NOCT, CRY1, DRD2, DRD3, LGR4, CIART |
| 37 | GO:0043666 | regulation of phosphoprotein phosphatase activity | 3 | 0.02967 | PPP6R1, TSC1, HSP90B1 |
| 38 | GO:0030212 | hyaluronan metabolic process | 3 | 0.02967 | ITIH4, ITIH3, ITIH1 |
| 39 | IPR003350 | Homeodomain protein CUT | 3 | 0.03469 | CUX2, CUX1, SATB2 |
| 40 | GO:0015180 | L-alanine transmembrane transporter activity | 3 | 0.03565 | SLC36A1, SLC36A2, SLC36A4 |
| 41 | GO:0009881 | photoreceptor activity | 3 | 0.03565 | OPN1SW, RHO, RRH |
| 42 | IPR017970 | Homeobox, conserved site | 12 | 0.03706 | BARHL1, ZFHX3, CUX2, PROP1, CDX2, HOXC5, HOXC4, HOXC9, HOXC8, HOXC11, PBX1, PITX3 |
| 43 | oas04145 | Phagosome | 13 | 0.03778 | ATP6V1G1, TFRC, NCF2, STX18, FCAR, DYNC1LI2, PIKFYVE, CLEC7A, MRC1, OLR1, SEC61B, DYNC1I1, ATP6V1F |
| 44 | GO:0035861 | site of double-strand break | 5 | 0.03806 | RNF169, SLF2, RAD51, APLF, NBN |
| 45 | GO:0005509 | calcium ion binding | 36 | 0.03831 | CRB1, USP32, STAB2, EFCAB3, CIB1, ATP2A1, NOL3, MMP20, DLL4, CAPN8, MMP27, CDH1, CAPN2, CHP1, PLA2G12A, S100A2, SYT1, MMP1, ANXA4, MMP3, TBC1D8, ANXA11, MMP8, MMP12, MYL2, MATN1, FAT2, S100A5, FAM20C, CASQ1, CDH16, MEGF8, MATN3, S100A8, MATN2, SLC25A13 |
| 46 | SM01109 | SM01109 | 3 | 0.04035 | CUX2, CUX1, SATB2 |
| 47 | GO:0004222 | metalloendopeptidase activity | 11 | 0.04054 | MMP12, ADAMTS14, MMP27, MMP1, PAPPA, ADAM12, MMP3, PMPCA, MMP8, MMP20, PAPPA2 |
| 48 | GO:0043565 | sequence-specific DNA binding | 20 | 0.04094 | BARHL1, NFE2, ZFHX3, ZGPAT, PROP1, ZBTB49, SATB2, CREBL2, NR3C1, ELF2, CUX1, HOXC5, ERF, HSF4, HOXC4, HOXC9, HOXC8, ATF6, PITX3, RAG1 |
| 49 | GO:0015824 | proline transport | 3 | 0.04282 | SLC36A1, SLC36A2, SLC36A4 |
| 50 | GO:0005788 | endoplasmic reticulum lumen | 5 | 0.04344 | ERLEC1, ERP29, MZB1, TXNDC16, HSP90B1 |
| 51 | GO:0007368 | determination of left/right symmetry | 6 | 0.04401 | DAAM2, DNAH5, DAW1, PCSK5, CC2D2A, BICC1 |
| 52 | GO:0005856 | cytoskeleton | 10 | 0.04514 | EPB41L4B, TEC, PRC1, EPB41, SNTG1, RIPK2, FRMPD1, HIP1R, PPL, PTPN3 |
| 53 | IPR001664 | Intermediate filament protein | 8 | 0.04597 | K38, KRT15, KRT35, KRT34, K33, V15, KRT32, KRT9 |
| 54 | IPR020479 | Homeodomain, metazoa | 8 | 0.04597 | BARHL1, CDX2, HOXC5, HOXC4, HOXC9, HOXC8, HOXC11, HOXC10 |
| 55 | GO:0005737 | cytoplasm | 123 | 0.04933 | SLC23A2, CRTC3, SLC23A1, STMN3, TTF2, NR3C1, SMC4, PPP1CB, SESN1, SPATA24, NUSAP1, MEIOC, KNTC1, KPNA4, OIP5, TNS3, CD96, USP3, PPP4R3B, CDC25C, FBXO10, HCFC2, ZFP42, PRKCQ, UTRN, BLM, GGNBP2, EPB41, TXK, SPATA2, SPATA5, LIN7C, STRIP2, MAP2, SNTG1, MICAL1, XRRA1, UBR5, IVNS1ABP, EGR1, ZFHX3, TMEM86B, SRMS, KLHL25, PLK1, STYX, IFT122, KLHDC8B, PTK6, PBX1, QKI, NMRAL1, NFKB2, GDF9, GRHPR, QARS, CCDC88C, RNF182, NF1, NDUFAF1, GMEB2, CPQ, TRADD, CDCA7L, C22H10ORF90, STAB2, OTUD7B, TFCP2L1, SMG7, HOXC11, EPB41L4B, CA1, PPP6R1, NTMT1, TRIM2, ARIH2, RIPK2, RIPK4, RHOH, GFRA3, WDR35, ARID1B, DUSP6, MLF1, ARL4C, DDB1, ELF2, PEAK1, IRF5, BCAT1, RCCD1, MAPRE2, PAFAH1B3, KIZ, ITIH4, PCNA, NAA20, SATB2, ZBTB49, ATP1A2, EPS8L1, SH3BP4, FLNC, HNRNPA1, PAIP2, TRIM45, KDM4A, NOP58, EYA1, SMOX, KLHL2, PTPN11, MYO19, WEE1, NLRP12, PSMC6, BRE, PRC1, NEURL1, RHEBL1, PPP1R14D, SNTB2, PTPN3 |
